# Neurovascular Uncoupling in Schizophrenia: A Bimodal Meta-Analysis of Brain Perfusion and Glucose Metabolism

**DOI:** 10.3389/fpsyt.2020.00754

**Published:** 2020-08-05

**Authors:** Niron Sukumar, Priyadharshini Sabesan, Udunna Anazodo, Lena Palaniyappan

**Affiliations:** ^1^ Department of Psychiatry, University of Western Ontario, London, ON, Canada; ^2^ Lawson Health Research Institute, London, ON, Canada; ^3^ Department of Medical Biophysics, Western University, London, ON, Canada; ^4^ Robarts Research Institute, University of Western Ontario, London, ON, Canada

**Keywords:** schizophrenia, arterial spin labeling, positron emission tomography, cerebral blood flow, cerebral glucose metabolism, dorsal anterior cingulate cortex

## Abstract

The use of modern neuroimaging approaches has demonstrated resting-state regional cerebral blood flow (rCBF) to be tightly coupled to resting cerebral glucose metabolism (rCMRglu) in healthy brains. In schizophrenia, several lines of evidence point toward aberrant neurovascular coupling, especially in the prefrontal regions. To investigate this, we used Signed Differential Mapping to undertake a voxel-based bimodal meta-analysis examining the relationship between rCBF and rCMRglu in schizophrenia, as measured by arterial spin labeling (ASL) and ^18^Flurodeoxyglucose positron emission tomography (FDG-PET) respectively. We used 19 studies comprised of data from 557 patients and 584 controls. Our results suggest that several key regions implicated in the pathophysiology of schizophrenia such as the frontoinsular cortex, dorsal ACC, putamen, and temporal pole show conjoint metabolic and perfusion abnormalities in patients. In contrast, discordance between metabolism and perfusion were seen in superior frontal gyrus and cerebellum, indicating that factors contributing to neurovascular uncoupling (e.g. inflammation, mitochondrial dysfunction, oxidative stress) are likely operates at these loci. Studies enrolling patients on high doses of antipsychotics had showed larger rCBF/rCMRglu effects in patients in the left dorsal striatum. Hybrid ASL-PET studies focusing on these regions could confirm our proposition regarding neurovascular uncoupling at superior frontal gyrus in schizophrenia.

## Introduction

Since the time of Ernst von Feuchtersleben who coined the term psychosis in 1845 (1), psychotic disorders have been suspected to be associated with disturbances in the cerebral blood supply. This has been thoroughly investigated through the use of modern neuroimaging techniques, which have uncovered abnormalities in the resting-state regional cerebral blood flow (rCBF) across various brain regions in schizophrenia. The frontal lobe, anterior cingulate cortex, temporal lobe and occipital lobe, among others, are regions that have been showed to differ with respect to rCBF in patients compared to healthy subjects (2). In healthy brains, rCBF is tightly coupled to resting cerebral glucose metabolism (rCMRglu), which increases with synaptic activity. This coupling, also known as functional hyperemia, is accomplished by the coordinated activity of a group of cells (comprised of astrocytes, endothelial cells, and neurons) called the neurovascular unit. These cells detect changes in synaptic activity, and initiate vasodilation or vasoconstriction responses to accommodate for the resultant changes in rCMRglu (3).

Two imaging modalities that have been very useful in studies investigating rCBF and rCMRglu in patients are arterial spin labeling (ASL), and positron emission tomography (PET). ASL is a relatively recent neuroimaging modality that was developed as a non-invasive analog to gadolinium contrast MRI for the measurement of rCBF. Instead of using a potentially toxic contrast to visualize blood flow, a radiofrequency pulse is applied at the neck region to magnetize blood water molecules flowing into the brain. This allows for the capturing of a “tagged” image in the area of interest by MRI. By quantitatively comparing the tagged image with a (non-RF pulse) control image, researchers can construct an accurate representation of cerebral blood flow (4). Likewise, an accurate representation of rCMRglu can be constructed using PET neuroimaging. PET is a functional imaging technique that uses a radioactive tracer to measure the regional activity of the biological molecule that the tracer is attached to. A common tracer is ^18^flurodeoxyglucose (FDG), and it is often employed in neuroimaging studies to measure the cerebral metabolic rate of glucose (5). ASL and FDG PET imaging have been used in various case-control studies to quantify case-control differences between patients with schizophrenia and healthy controls.

The vascular hypothesis of schizophrenia suggests that one of the underlying mechanisms of schizophrenia is the disruption of the appropriate rCBF response to changes in cerebral metabolic activity (6). In healthy brains, the integrity of the neurovascular unit is essential in maintaining functional hyperemia and ensuring that changes in rCBF are tightly and congruently coupled to changes in rCMRglu. Disruption of this coupling (low rCBF with high rCMRGlu) can lead to insufficient support for synaptic activity, triggering synaptic loss or promoting glia-mediated inflammatory response that can result in a cascade of further damage to the synaptic and neuronal homeostasis required for intact cerebral function (7). Neurovascular uncoupling can also impair oxygen metabolism, induce mitochondrial dysfunction and oxidative stress leading to neuronal death, and brain tissue atrophy (8).

Identifying regions where this uncoupling occurs is extremely important as the underlying mechanism and its pathophysiological relationship to schizophrenia can be studied in more detail. This has been done to some degree; uncoupling has been demonstrated to occur in patients with schizophrenia, especially in the prefrontal regions during task-related activities (9). However, no simultaneous ASL-PET studies identifying regions with concordance or discordance between metabolism and perfusion have been reported to our knowledge. To address this gap, we undertook a voxel-based bimodal meta-analysis to examine the relationship between rCBF and rCMRglu in schizophrenia. We hypothesized that several brain regions would show combined abnormalities of perfusion and metabolism, while uncoupling of these two parameters would be observed in prefrontal regions. The meta-analysis was performed using the anisotropic effect size version of seed-based d mapping (AES-SDM). AES-SDM is a software used to create meta-analytic maps of studies that use MNI or Talairach coordinates to denote brain regions of significant group differences, weighted by sample size, variance, and between-study heterogeneity. AES-SDM was also used to conduct meta-regression analyses to quantify the effect of nuisance variables such as age, gender, duration of illness, antipsychotic dosage, and illness severity on the heterogeneity of the findings. It is important to note that the absolute values of the outcome measures in various studies differ based on the quantification procedures employed in both ASL and PET studies. Our meta-analysis is based on effect-sizes of differences between two groups, rather than the absolute measurement metrics. As such, this study is an early probe to assess the likelihood of regions with neurovascular uncoupling in schizophrenia.

## Methods

### Search

**Figure 1:**
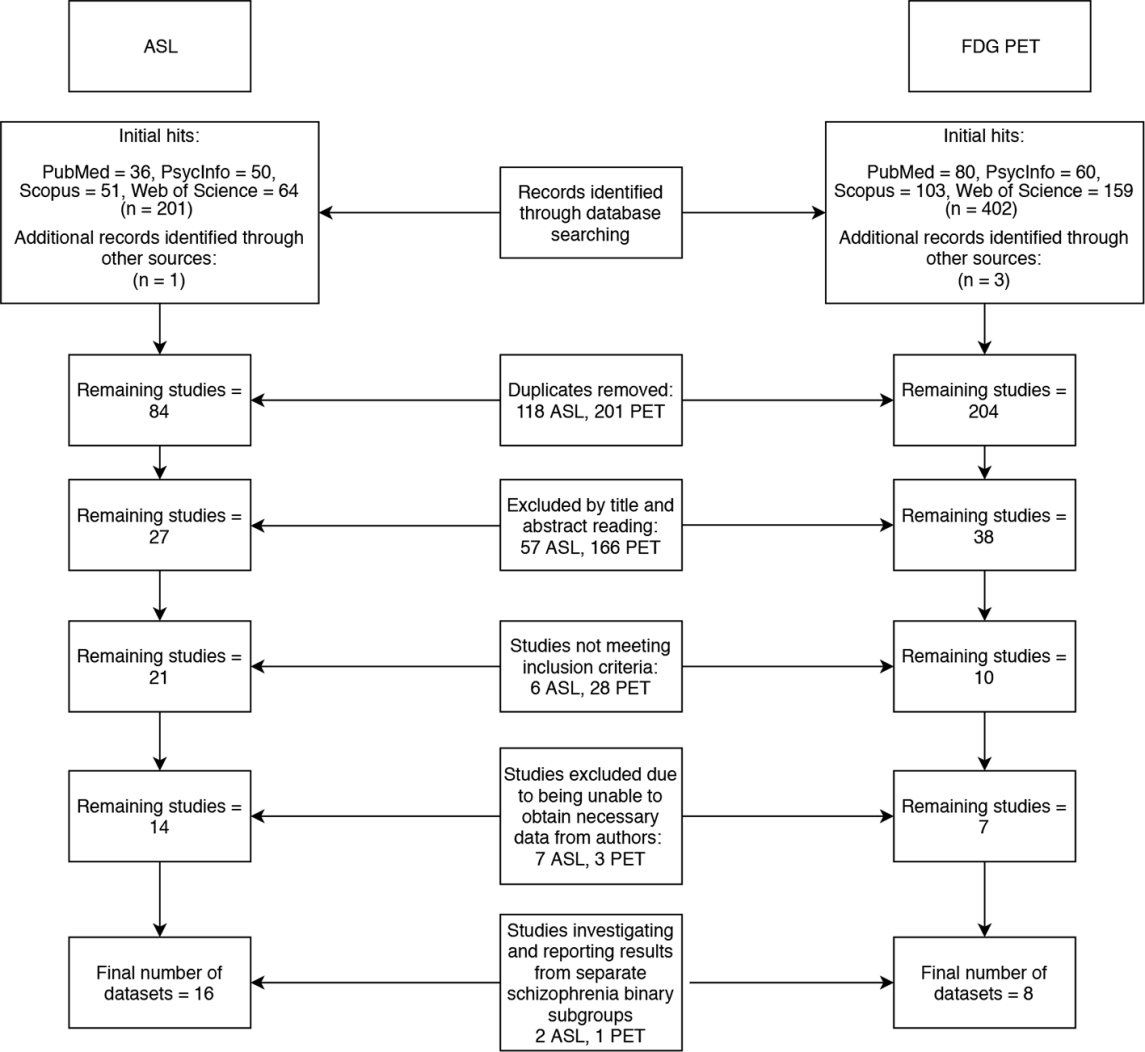
Flowchart of literature search.

Two literature searches were conducted across four databases (PubMed, PsycInfo, Scopus and Web of Science). The search terms “arterial,” “spin,” “labeling,” and “schizophrenia” yielded 83 results, and the terms “FDG,” “PET” and ‘schizophrenia’ yielded 201 results (after duplicates were removed). The following inclusion criteria was applied: Case control studies reporting voxelwise ASL or FDG-PET changes in patients with schizophrenia compared to healthy controls, using MNI or Talairach coordinates. Studies that met the inclusion criteria, but did not report all of the data required for the meta-analysis were not immediately excluded in the hope that a correspondence with the authors of these studies could be initiated to obtain the missing data. Studies that did not use ICD 10 or DSM IV/5 diagnostic criteria for schizophrenia or did not investigate the whole brain were excluded. Twenty-one ASL and eight PET papers remained after inclusion and exclusion criteria were applied to the search results. Of these, 12 papers did not report their data in a format that was compatible with our meta-analysis. We contacted the authors of these papers and were only able to obtain additional data from two studies (2, 10). Three of the papers we finally included in our meta-analyses (11–13), investigated schizophrenia binary subgroups (for example, catatonic and non-catatonic schizophrenia patients) compared to controls. The final meta-analyses thus used 16 datasets from 14 ASL papers (2, 10–12, 14–23) and 6 datasets from 5 PET papers (13, 24–27) to generate results. The literature search is summarized in the flowchart in **Figure 1**.

### ASL Study Quality

The methodological quality of the included studies was assessed by scoring four criteria encompassing all aspects of data collection and analysis which can confound the quality of the ASL data and subsequently bias the validity and reliability of the study inferences. The four criteria are: (1) participant selection, (2) image acquisition, (4) image preprocessing and analysis, and (5) statistical analysis techniques. The criteria for appropriate quality standards for image acquisition were based on the recommended implementations of ASL by the ISMRM Perfusion Group and the European ASL in Dementia (28). The minimum standard requirement for image acquisition were modified since 36% of the included ASL studies were completed 2–3 years after the recommended guidelines. Emphasis was placed on determining whether the included studies specified imaging parameters that impact the signal-to-noise in ASL images. ASL image quality increases as the signal-to-noise increases. The criteria were scored as either “adequate,” if all aspects of the criteria were reported and met minimum standard, or “inadequate,” if aspects of the criteria were missing or did not meet minimum standard, or “unclear,” if no clear conclusion could be drawn from the information provided. These criteria are further detailed in **Table 1**.

**Table 1:** Quality analysis tool for included arterial spin labeling (ASL) studies.

	Criteria	Minimum standard
1	Participant Selection	Participants in the study should be subjects with a diagnosis of schizophrenia or schizoaffective disorder established through clinical chart review using structured diagnostic interviews based on DSM-IV/V and healthy controls between ages of 18 and 65 years. Participants were selected following a prospective inclusion and exclusion criteria. Details should be provided about age distribution, female to male ratio and disease description as minimum information (subject demographics table). Total number of subjects per group were >10.
2	Image acquisition	*Emphasis placed on factors that impact signal-to noise.* Hardware considerations: Scanner manufacturer and Field Strength specified was 1.5 T or 3T. The specific ASL pulse labeling approach was specified. The four labeling approaches are: continuous (CASL), pseudo-continuous (pCASL), pulsed (PASL), and velocity-selective (VS-ASL). Time between labeling and imaging (post-label delay) was specified Total imaging time was specified, or enough information was specified to estimate total imaging time (repetition time, echo time, and the number of label and control pairs).
3	Image analysis	Measures to restrict motion or correct motion was specified. ASL is sensitive to motion and motion can reduce signal-to-noise in ASL. The following parameters used in calculating CBF must be specified: The blood-brain partition coefficient used The T1 of blood used The Labeling efficiency used The use of a brain atlas or template for spatial normalization was specified.
4	Statistical analysis	Statistical methods for controlling multiple comparisons were specified for significant differences. The influence of age and gender was controlled/removed. ** *Age and gender are significant CBF modifiers (Clement et al. JCBFM 2017)*

Each criterion was categorized as follows: Adequate: All aspects of the criteria were reported and met minimum standard. Inadequate: Aspects of the criteria were missing or did not meet minimum standard. Unclear: No clear conclusion could be drawn from the information provided.

### PET Study Quality

The methodological quality of the included studies was assessed by scoring five criteria encompassing all aspects of data collection and analysis which can confound the quality of the PET data and subsequently bias the validity and reliability of the study inferences. The five criteria are (1) participant selection, (2) participant preparation, (3) image acquisition, (4) image preprocessing and analysis, and (5) statistical analysis techniques. The criteria for appropriate quality standards for participant preparation and image acquisition were based on the SNMMI Procedure guideline for FDG PET Brain Imaging Version 1.0 (29). The criteria were scored as either “adequate,” if all aspects of the criteria were reported and met minimum standard, or “inadequate,” if aspects of the criteria were missing or did not meet minimum standard, or “unclear,” if no clear conclusion could be drawn from the information provided. These criteria are further detailed in **Table 2**.

**Table 2:** Quality assessment tool for included positron emission tomography (PET) studies.

	Criteria	Minimum standard
1	Participant Selection	Participants in the study should be subjects with a diagnosis of schizophrenia or schizoaffective disorder established through clinical chart review using structured diagnostic interviews based on DSM-IV/V and healthy controls between ages of 18 and 65 years. Participants were selected following a prospective inclusion and exclusion criteria. Details should be provided about age distribution, female to male ratio and disease description as minimum information (subject demographics table). Total number of subjects per group were >10.
2	Participant preparation	Participants fasted for 4–6 hours. The use of caffeine or alcohol, substance use/abuse/dependence, or psychoactive medications that may affect cerebral glucose metabolism were specified. The blood glucose was checked prior to FDG-injection and was no greater than 150 – 200 mg/dl. Participants were kept in a stable environment during PET uptake period. This includes a quiet, dimly-lit room and eyes open/closed.
3	Image acquisition	Scanner make and model was specified. Administered FDG dose was within 185–740 Mbq Uptake period within 20–60 min Emission scan duration of a minimum of 15 min
4	Image analysis	An attenuation correction method was specified. The reconstruction algorithm was specified. Measures to restrict motion or correct motion was specified. The use of a brain atlas or template for spatial normalization was specified. An appropriate reference region selection (cerebellar cortex or whole brain) for count normalization was reported.
5	Statistical analysis	Exclusion of none gray matter voxels was reported. Statistical methods were specified for significant differences The influence of age and gender was controlled/removed.

Each criterion was categorized as follows: Adequate: All aspects of the criteria were reported and met minimum standard. Inadequate: Aspects of the criteria were missing or did not meet minimum standard. Unclear: No clear conclusion could be drawn from the information provided.

### Mean Analysis

AES-SDM was used to generate a meta-analytic map for the compiled datasets, using permutation tests to determine statistical significance of results. As recommended by Radua et al., default SDM parameters were used (FWMH = 20mm, cluster extent = 10 voxels), but with a more conservative uncorrected p-value (P < 0.001 compared to P < 0.005) (30). We assessed the robustness of the results by assessing the residual heterogeneity, and by using a sensitivity analysis (jack-knife approach). This was done by repeating the meta-analysis multiple times, each time leaving out one of the studies that was originally included it. A score was given to each reported brain region corresponding to the number of times it was reported in the meta-analyses. In addition, we examined the peaks of maximum heterogeneity to check if they are driven by a small number of “outliers.” We also drew funnel plots for each peak voxel reported in the main analysis using the Bias Analysis option of the SDM (version 6.21) and statistically assessed for asymmetry in the funnel plot (using ‘metabias’ function of R (31)) and a test for publication bias favoring small studies with large effect sizes (based on (32), implemented *via* ‘MetaNSUE’ function of R (33)).

### Conjunction Analysis of rCBF and rCMRglu Changes

We used multimodal analysis to identify which of the brain regions identified in our meta-analysis showed significant changes in both rCBF and rCMRglu. Our goal was not necessarily to demonstrate a physiological correlation between these measures, but rather to identify which of our results were supported by data from both ASL and PET imaging studies. As this was a four-tailed test (allowing for positive and negative results across two modalities), we used a conservative threshold of p<0.0025 for cluster inclusion and p<0.00025 (10-times more stringent) for peak identification, based on the minimum acceptable threshold for conjunction in each individual modality map as p<0.05. This method, described in detail by Radua et al. (34), has been used in various studies for bimodal conjunction meta-analysis (35–37). We used MRIcron software to generate visual representations of the findings.

### Disjunction Analysis of rCBF and rCMRglu Changes

We used the meta-regression tool to identify which of the brain regions identified in our meta-analysis showed significant disjoint or “uncoupled” changes in rCBF and rCMRglu. We assigned a binary value to each study corresponding to the measure being investigated (rCBF - 0; rCMRglu - 1) and tested the slope (1m0) of the effect of the measure (ASL vs PET) on effect-sizes reported in the SDM. A significant positive regression value for a given brain region indicates an uncoupled increase in rCMRglu or an uncoupled decrease in rCBF, and a significant negative regression value indicates an uncoupled decrease in rCMRglu or an uncoupled increase in rCBF. In order to determine the appropriate case, each of the results of the regression analysis were cross-referenced to the brain regions identified by the primary meta-analysis. We used MRIcron software to generate visual representations of the findings.

### Meta-Regression Analysis

We explored the influence of age, gender, duration of illness, PANSS positive, and negative symptom severity, and overall dose of antipsychotic medications (in chlorpromazine equivalents) on the reported effect-sizes. To reduce spurious relationships, in line with prior studies (36) we used a probability threshold of 0.0005, and tested the slope (1m0) (e.g. comparing effect sizes in studies with lowest vs. highest values of the predictor variable of interest), and restricted meta-regression to findings detected in the main analyses (38). We performed a separate meta-regression for each of the variables being investigated. We also visually inspected the regression plots from peak coordinates and discarded slopes driven by <5 studies (30).

### Querying Functional Connectivity Differences

The peak coordinates of various regions of interest identified by the conjunction and disjunction analysis were used to determine the brain network connectivity of these regions. This was done using the Neurosynth functional connectivity meta-analytical database.

## Results

### Study Demographics

Our meta-analysis drew data from 22 datasets from 19 studies. In total, 557 patients with schizophrenia were compared to 584 healthy controls. **Table 3** lists the demographic data for the participants in each study. Most studies published information regarding number of patients and healthy controls, patient gender distribution, patient age, duration of illness, antipsychotic dosage, syndrome severity, type of scan, and study quality. Almost all of the studies matched healthy controls to patients for at least age and gender, with some studies matching controls for other variables such as level of education. Duration of illness, antipsychotic dosage and PANSS scores ranged from 25.2 (SD 5.58) to 92.0 (SD 22) for ASL studies and from 33.8 (SD 5.3) to 84.7 (SD 23.6) for PET studies. With a few exceptions, all included ASL studies used a 3T scanner with a pCASL technique to obtain results and only included patients diagnosed with schizophrenia specifically. One study (14) used a pASL technique, one study (15) used a 1.5T MRI scanner and three studies (10, 16, 17), included individuals with schizoaffective disorder in their patient groups. All PET studies only included patients diagnosed with schizophrenia disorder specifically. The authors from studies with missing clinical information were contacted, and some of the unpublished data was retrieved for this review.

**Table 3:** Demographic table for datasets included in meta-analysis, as well as the quality scores for each study.

Study	Modality	Patients	Controls	Patient Age	Control Age	% Females	PANSS	PANSS Positive	PANSS Negative	Duration of Illness	CLPZ eq.	Quality scores
**Kindler 2018**	ASL	32	31	41.6 ± 13.4	39.4 ± 12.3	43.75	76.6 ± 17.4	19.6 ± 6.7	19.7 ± 6.0	14.3	497 ± 210	0.8
**Walther 2017 (Catatonia)**	ASL	15	41	35.9 ± 12.7	38.6 ± 13.6	26.67	77.1 ± 18.9	16.0 ± 8.1	21.9 ± 6.9	12.8 ± 12.0	461.3 ± 346.4	0.53
**Walther 2017 (Non-Catatonia)**	ASL	27	41	37.1 ± 10.6	38.6 ± 13.6	37.04	67.1 ± 16.3	17.9 ± 6.2	16.4 ± 4.1	10.5 ± 10.6	373.6 ± 359.4	0.53
**Pinkham 2015 (Paranoid)**	ASL	16	25	38.5 ± 7.71	33.64 ± 12.42	50	32 ± 5.37	19.19 ± 3.53	32.0 ± 5.37	NA	332.2 ± 546.75	0.93
**Pinkham 2015 (Non-Paranoid)**	ASL	16	25	38.8 ± 13.24	33.64 ± 12.42	31.25	25.2 ±5.58	12.63 ± 3.76	25.19 ± 5.58	NA	350.5 ± 571.26	0.93
**Zhu 2015**	ASL	100	94	33.6 ± 8.6	33.3 ± 10.4	43	71.3 ± 22.7	17.0 ± 7.8	20.1 ± 9.0	10.2 ± 8.2	453.2 ± 342.9	0.73
**Kindler 2015**	ASL	34	27	41.5 ± 12.9	39.8 ± 12.6	47.06	75.5 ± 17.3	19.9 ± 6.8	18.9 ± 6.1	NA	518.9 ± 235.7	0.73
**Ota 2014**	ASL	36	42	37.9 ± 12.6	37.9 ± 13.0	52.78	61.8 ± 19.3	15.3 ± 5.8	14.9 ± 6.2	16.8 ± 11.3	604.8 ± 459.2	0.73
**Pinkham 2011**	ASL	30	24	35.7	35.73	40	NA	NA	NA	NA	373.7	0.93
**Walther 2011**	ASL	11	14	35.3 ± 12.54	31.71 ± 6.08	27.27	54.2 ± 14.11	11.73 ± 4.45	18.00 ± 7.59	8.9 ± 13.29	442.5 ± 241.03	1.00
**Scheef 2010**	ASL	11	25	32	30	27.27	43.1 ± 8.5	20.2 ± 2.9	21.4 ± 7.9	NA	NA	0.93
**Horn 2009**	ASL	13	13	29.6 ± 11.2	26.6 ± 4.6	38.46	63.9	13.48 ± 4.8	15.5 ± 6.1	2.79 ± 2.6	556.2	0.87
**Oliveira 2018**	ASL	28	28	32.8 ± 7.8	31.1 ± 5.8	14.29	69.4 ± 17.2	14.4 ± 6.4	23.1 ± 8.2	14.3	546.4	1.00
**Cui 2017 (Non-AVH)**	ASL	25	25	24 ± 5	26 ± 5	48	92 ± 22	21 ± 9	23 ± 10	1.5 ± 1.75	NA	0.87
**Stegmayer 2017**	ASL	20	30	38.2 ± 11.4	36.7 ± 12.9	61.7	72.6 ± 17.1	18.2 ± 6.4	18.4 ± 5.1	12.2 ± 12.3	400.2 ± 344.2	1.00
**Liu 2012**	ASL	19	20	NA	NA	57.90	NA	NA	NA	20.5 ± 10.0	622.1 ± 418.0	0.67
**Park 2009**	PET	29	21	29.8 ± 3.9	3.9 ± 3.0	48.27	33.8 ± 4.8	15.9 ± 3.0	16.7 ± 2.5	7.5 ± 4.1	633.2 ± 415.6	0.65
**Desco 2002**	PET	51	18	31.93	13.16	31.37	NA	NA	NA	7.89	NA	0.6
**Kim 2017**	PET	19	18	32.6 ± 12.0	30.7 ± 7.9	36.84	84.7 ± 23.6	NA	NA	2.3 ± 1.8	NA	0.75
**Ben-Shachar 2006 (HPS)**	PET	8	8	35.9 ± 11.8	34.5 ± 11.5	37.5	43.7 ± 6.4	24.5 ± 6.4	23.2 ± 6.8	15.57 ± 11.3	NA	0.65
**Ben-Shachar 2006 (LPS)**	PET	8	8	42.7 ± 15.3	34.5 ± 11.5	50	33.6 ± 5.3	11.0 ± 5.3	19.7 ± 7.6	18.42 ± 10.3	NA	0.65
**Horga 2014**	PET	9	8	NA	NA	NA	45 ± 8.2	26.33 ± 4.15	20.66 ± 5.44	2.1 ± 7.8	NA	0.8

Age and duration of illness are given in years.

### Study Quality

**Figure 2:**
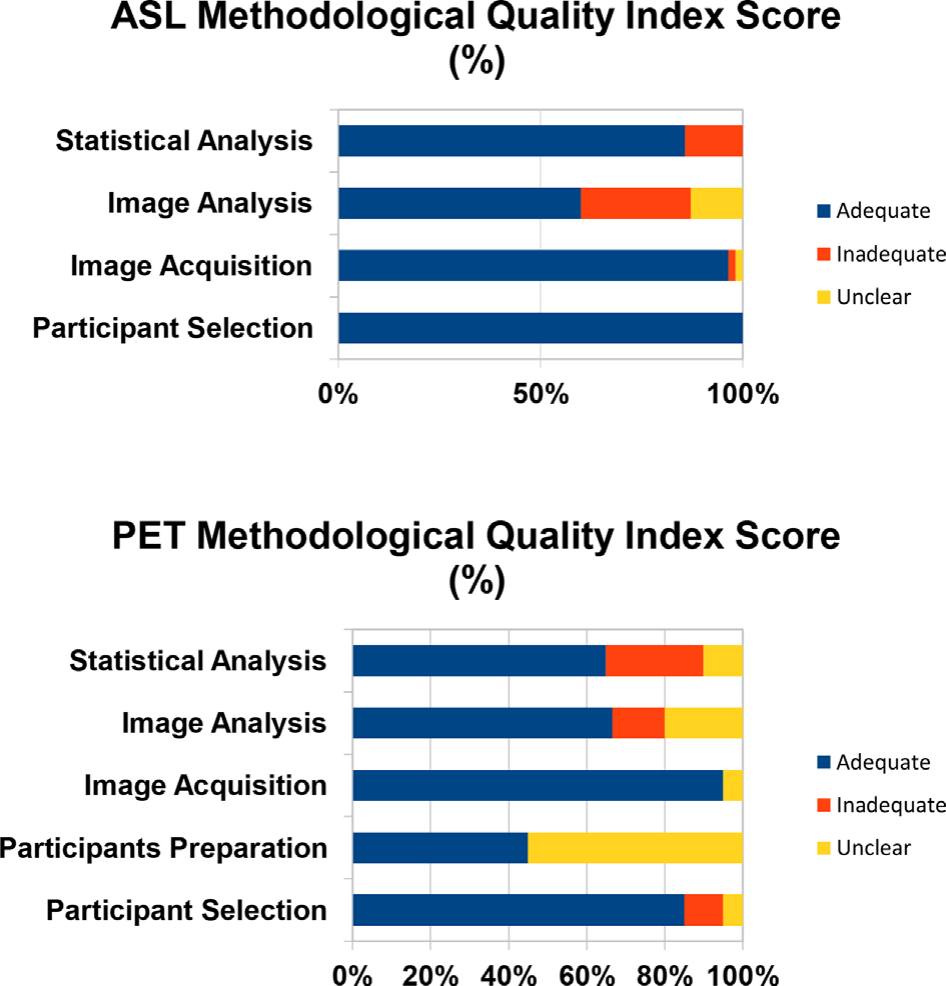
The Quality Index Score is the proportion of studies that are scored as adequate, inadequate, or unclear.

The average quality scores for the ASL and PET studies used in the meta-analysis were 0.84 and 0.69 respectively. Among the ASL studies, the lowest quality score was 0.53 (11) and among the PET studies the lowest quality score was 0.6 (13). The Quality Index Scores for these studies are visually represented in **Figure 2**.

### Mean Analysis

The SDM analysis showed several brain regions of significant difference in rCBF or rCMRglu between patients and healthy controls. These results are listed in **Table 4**. Patients had significantly increased neurological activity in the right lenticular nucleus, left striatum, right inferior temporal gyrus, left temporal pole, right thalamus, and corpus callosum. Patients had significantly reduced neurological activity in the right median cingulate, right middle occipital gyrus, left inferior frontal gyrus, left superior occipital gyrus, and right superior frontal gyrus. Other than the right superior frontal gyrus, which only survived 17 cross-validations, each of the other regions survived at least 21 cross-validations. None of the reported peaks had asymmetric funnel plot, or excess significance bias (**Table 5**). Moderate heterogeneity (I^2^>40%) was noted for left striatum, left temporal pole, right anterior thalamic projections, and left inferior frontal gyrus, while all other regions had low levels of heterogeneity.

**Table 4:** Brain regions of significant difference in regional cerebral blood flow (rCBF) or resting cerebral glucose metabolism (rCMRglu) between patients with schizophrenia and controls.

Region	SDM Z	MNI Coordinate	Cluster Size	P value	Jack-knife Score	Cluster breakdown	
**Patients>controls**						
Blobs of ≥ 59 voxels with all voxels SDM-Z ≥ 1.655 and all peaks SDM-Z ≥ 1.982						
**Right Putamen**	3.517	28,4,12	1411	<0.0001	22	BA48 BA34	
**Left Striatum**	3.186	−22,2,6	1080	<0.0001	22	BA48 BA34	
**Right Inferior Temporal Gyrus**	3.186	42,2,-42	596	<0.0001	22	BA20 BA36	
**Left Temporal Pole**	2.470	−34,0,−44	295	0.0005	21	BA20 BA36	
**Right Anterior Thalamic Projections**	2.204	18,–22,12	103	0.0005	21		
**Corpus Callosum**	1.982	18,−28,4	59	0.001	21	n/a	
**Controls>patients** Blobs of ≥ 101 voxels with all voxels SDM-Z ≤ –1.894 and all peaks SDM-Z ≤ –2.218						
**Right Median Cingulate Gyrus**	−2.829	8,26,32	1292	0.0005	22	BA24 BA32	
**Right Middle Occipital Gyrus**	−2.873	30,−92,12	357	<0.0001	21	BA18 BA19	
**Left Superior Occipital Gyrus**	−2.641	−16,−100,14	274	<0.0001	22	BA17 BA18	
**Left Inferior Frontal Gyrus, (Triangular)**	−2.708	−42,22,-2	647	<0.0001	21	BA47 BA45 BA48	
**Right Superior Frontal Gyrus, (Dorsolateral)**	−2.218	20,20,56	101	0.001	17	BA8	

Jack-knife analysis was scored out of 22. Voxel threshold: P < 0.005; Peak height threshold: peak SDM-Z > 1.000; Extent threshold: cluster size ≥ 10 voxels.

**Table 5:** Estimates of between-study heterogeneity and publication bias from peak regions of significant difference in regional cerebral blood flow (rCBF) or resting cerebral glucose metabolism (rCMRglu) between patients with schizophrenia and controls.

Region	MetaBias test(z/p value)	Excess significance test (p value)	I^2^ statistic
**(Regions showing increased rCBF/CMRglu in patients)**			
**Right Putamen**	z: −0.67, p=0.502	0.86	3.47
**Left Striatum**	NA	0.376	50.54
**Right Inferior Temporal Gyrus**	Z: −0.94, P = 0.349	0.842	7.37
**Left Temporal Pole**	Z: −0.29, P = 0.829	0.897	45.49
**Right Anterior Thalamic Projections**	Z: −1.41, P = 0.304	0.937	41.44
**Corpus Callosum**	Z: −0.75, P = 0.453	0.894	1.74
**(Regions showing decreased rCBF/CMRglu in patients)**			
**Right Median Cingulate Gyrus**	Z: 0.62, P = 0.538	0.492	30.1
**Right Middle Occipital Gyrus**	Z: 0.16, P = 0.873	0.671	2.21
**Left Superior Occipital Gyrus**	NA	NA	15.1
**Left Inferior Frontal Gyrus, (Triangular)**	Z: 0.48, P = 0.632	0.895	44.1
**Right Superior Frontal Gyrus, (Dorsolateral)**	Z: 0.09, P = 0.932	0.975	31.6

These results are obtained using Bias Test function of the Signed Differential Mapping software version 6.21.

### Conjunction and Disjunction Analyses of rCBF and rCMRglu Changes

**Figure 3:**
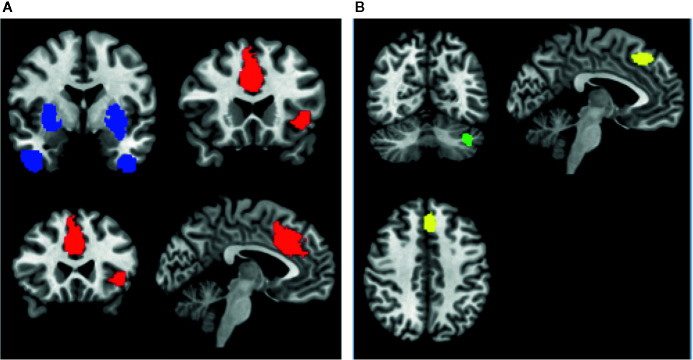
Regions of conjoint findings are shown on the left **(A)**, and regions with disjoint findings are shown on the right **(B)**. Bilateral striatum and temporal pole were found to have conjoint increases in regional cerebral blood flow (rCBF) and resting cerebral glucose metabolism (rCMRglu) (shown in blue). Left frontoinsular cortex and bilateral dorsal anterior cingulate cortex were found to have conjoint reductions in rCBF and rCMRglu. Regional neurovascular uncoupling was notable in the left superior frontal gyrus (−6,30,44; Left BA 8; SDM-z = −2.001, p=0.00033, reduced rCMRglu, normal rCBF – shown in yellow) and left cerebellum (−38,−66,−34; crus I and II; SDM-Z=2.27, p=0.00026, no. of voxels = 129; increased rCMRglu, normal rCBF – shown in green).

Among patients with schizophrenia, we observed a conjoint reduction in rCBF and rCMRglu in the right median cingulate gyrus and left inferior frontal gyrus. A conjoint increase in rCBF and rCMRglu was noted in the right putamen and right inferior temporal gyrus. (Voxel probability threshold: p=0.0025, Peak height threshold: p=0.00025, Cluster extent threshold: 10 voxels). Regional neurovascular uncoupling was notable in the superior frontal gyrus (reduced rCMRglu, normal rCBF) and cerebellum (increased rCMRglu, normal rCBF). These regions are visually represented in **Figure 3**.

### Meta-Regression Analysis

Meta-regression analysis was conducted to investigate the relationship between the change in rCBF or rCMRglu in the regions identified by the meta-analysis and various nuisance variables of interest. Illness duration was found to be negatively correlated to rCBF/rCMRglu changes in the left inferior frontal gyrus. In addition, we found that higher antipsychotic dose exposure attenuated the effect size of the patient-control differences in right middle occipital and left striatal rCBF or rCMRglu. We also noted that lower negative symptom burden also related to attenuated effect size of the patient-control differences in medial occipital rCBF or rCMRglu. Interestingly, patient samples receiving lower average antipsychotic doses were reporting higher average PANSS negative symptom scores (r=−0.6, p=0.03, df=12; **Supplementary Figure S3**). The meta-regression findings are described in the supplementary data (**Table S1**).

### Querying Functional Connectivity Differences

**Figure 4:**
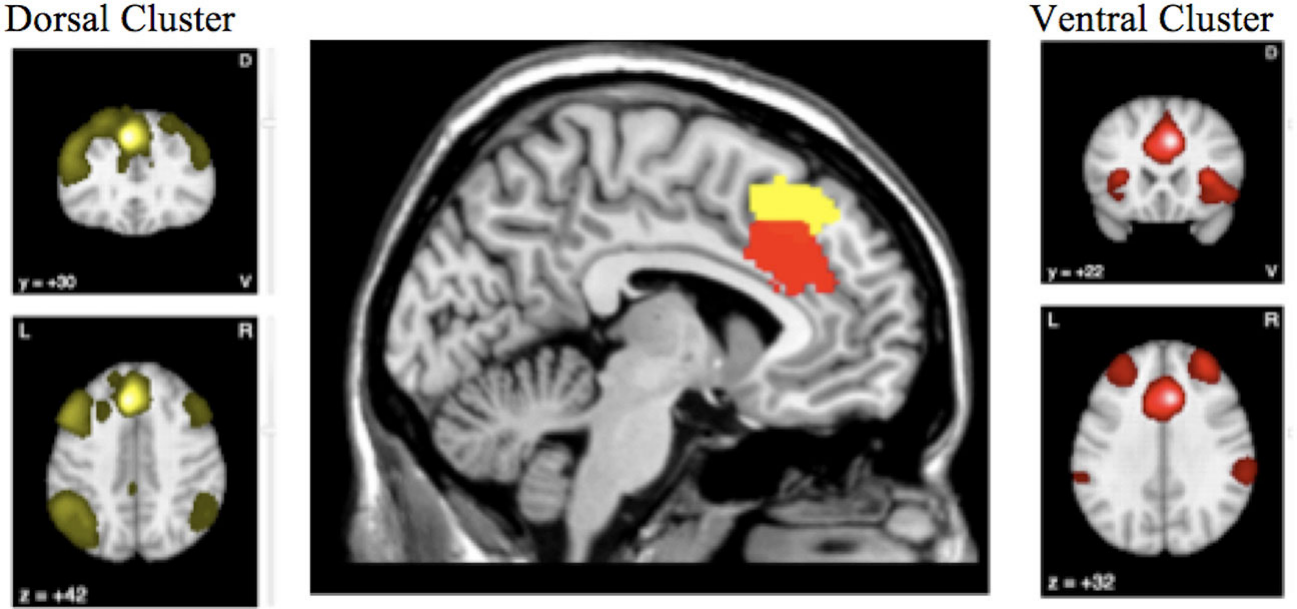
The conjunction and disjunction findings in the anterior cingulate region are shown. The more ventral cluster (red; 6, 22, 34) has reduced resting cerebral glucose metabolism (rCMRglu) as well as reduced regional cerebral blood flow (rCBF), while the more dorsal (yellow; −6, 30, 44) has normal rCBF despite reduced metabolism.

**Figure 4** highlights the anterior cingulate region, which showed conjoint and disjoint findings from different brain networks. The ventral cluster had reduced rCMRglu as well as reduced rCBF and was found to be well connected to the Salience Network, while the more dorsal cluster had normal rCBF despite reduced metabolism, and seems to participate in the frontoparietal executive network.

## Discussion

To our knowledge, this is the first bimodal neuroimaging meta-analysis which combines information from whole brain FDG-PET studies investigating resting metabolic state, and ASL studies investigating the resting blood flow in schizophrenia. We report three major findings: 1. The frontoinsular cortex and the bilateral dorsal anterior cingulate cortex show reduced rCBF as well as rCMRglu in schizophrenia; 2. The bilateral dorsal striatum and the temporal pole show increased rCBF as well as rCMRglu in schizophrenia; 3. Brain regions with rCBF changes consistently show rCMRglu changes, but brain regions with rCMRglu changes are not always coupled with rCBF changes, especially in the superior frontal (dorsomedial ACC) and cerebellar cortices.

The frontoinsular cortex and bilateral dorsal anterior cingulate cortex, which showed reduced rCBF as well as rCMRglu in schizophrenia, belong to the Salience Network (SN) (39). The SN is considered to be a cognitive control network that performs the critical function of switching from introspective default-mode of brain activity to extrospective task-processing activity (40). Many prior studies have extensively investigated the connectivity within the SN and between the SN and other brain regions, and have highlighted the concentration of gray matter reduction in schizophrenia around the nodes of the SN (41). Our findings once again highlight the primary role that the SN plays in the diagnostic construct of schizophrenia (42). The neural basis of insula-related observations in fMRI studies have been considered with some caution given the vascular anatomy of this region (43). Our bimodal analysis establishes that defects in glucose utilization of SN nodes are a key aspect of the pathophysiology of schizophrenia. We also note that the frontoinsular cortex shows a trend for reduction in rCBF/rCMRglu in patients with longer duration of illness, indicating the possibility of a progressive defect in this key node.

Studies enrolling patients on higher doses of antipsychotics are associated with larger rCBF/rCMRglu effects in the left striatum. The effect of antipsychotics on striatal rCBF in schizophrenia has been studied extensively, and our results are highly consistent with the synthesis reported by Goozée et al. (44). Striatal D2 blockade seems to have a direct effect on increasing metabolism as well as blood flow of striatum, while other brain regions do not exhibit a relationship of similar magnitude. Increased rCBF/rCMRglu of temporal pole is consistent with medial temporal lobe pathology that has been reported in the literature (45–47). Given that the posited function of the temporal pole is one of emotion-perception binding (47), hypermetabolism in this region may be related to acute psychotic symptoms as shown by Crespo-Feccorro et al., though our meta-regression was not able to confirm this notion. Both lower dose exposure and higher negative symptom burden related to the exaggerated effect size of the patient-control differences in middle occipital rCBF/rCMRglu. These findings are intriguing in the context of recent observations implicating occipital hypometabolism as an early biomarker of anti–NMDA receptor encephalitis, a condition with a high propensity to present with psychotic symptoms (48, 49). Nevertheless, we did not find any regional associations for the PANSS positive symptom domain. Given the limited power of our meta-regression approach, we urge caution in interpreting these negative results.

We observed rCBF/rCMRglu uncoupling in two sites: dorsomedial prefrontal cortex and cerebellar vermis. In particular, a large body of fMRI studies indicate that the BOLD signal in the dorsomedial prefrontal cortex is reduced when control/conflict-processing tasks are performed by patients, compared to healthy subjects (50). Our current results indicate that these BOLD results are likely driven by reduced synaptic activity rather than a perfusion deficit in this region. The presence of normal rCBF in a site with reduced rCMRglu indicates a relative hyperperfusion, and resonates with the observation made by Taylor et al. (51) who demonstrated that ACC region demonstrates a relative hyperperfusion in subjects with schizophrenia.

Coupling of CMRglu and CBF appears to be linked to a vasoactive mechanism, as well as a structural factor related to capillary density (52). Postmortem studies in schizophrenia have not uncovered any notable vascular structural pathology to date, except for an increase in astrocytic end-feet in the prefrontal cortex shown by one study (53). In certain mitochondrial encephalopathies and lactic acidosis, uncoupling of rCBF and CMRglu occurs (54, 55), indicating that alterations in oxidative stress pathways may be relevant to our current observation in schizophrenia. In experimental models, neurovascular coupling is impaired in young rodents when redox imbalance is created by increasing intracellular generation of superoxide radicals (56). Glutathione, a major intracellular antioxidant that protects neuroglia from oxidative stress by disposing peroxides, is notably reduced in schizophrenia particularly in the medial prefrontal cortex (57), especially in patients with poor outcomes (58, 59). Taken together, our results support the likely existence of redox imbalance in the medial prefrontal cortex of patients with schizophrenia.

There are several limitations in this review that should be considered when interpreting results. Firstly, the cerebellar disjunction findings must be considered with caution as the quality of ASL signals from this region has been suboptimal in many studies. The lack of cerebellar coverage as well as the influence of ASL labeling site on posterior cerebral circulation may have influenced the reported disjunction. Secondly, all of the reported PET studies used CT-based anatomical registration, while ASL uses MR-based information. This might have influenced the exact location of peak coordinates, though the spatial smoothing used in SDM mitigates this to some extent. We also urge caution in interpreting the negative results from meta-regression (i.e. lack of age, gender and severity effects on metabolism) as none of the individual studies were powered to detect these relationships, and the meta-regression approach cannot deal with non-linear effects. Furthermore, we acknowledge that the meta-regression is exploratory as this analysis is likely to be underpowered. Similarly, disjunction results could also be driven by the well-known issue of false negative results from coordinates based meta-analyses (60). Only one ASL study employed 1.5T scanner (15), and our Jack-knife sensitivity analysis indicated that dropping this study did not alter the overall results. We did not use scanner strength as a marker of study quality as both patients and controls in such studies were scanned using the same instrument, obviating any measurement bias. Nevertheless, it is likely that the lower SNR might have contributed to publication bias, with only a few 1.5 T studies with positive results are reported in the literature. Finally, our results do not demonstrate that metabolic and vascular abnormalities are necessarily correlated at the subject (or group) level. Our aim was more modest, and restricted to localizing those brain regions where both abnormalities coexist in schizophrenia.

To conclude, schizophrenia related regional perfusion abnormalities capture the aberrant metabolism of underlying neuro-glial tissue. In specific brain regions, such as the dorsomedial prefrontal cortex, neurovascular uncoupling suggestive of possible inflammation (causing inappropriate hyperemia), astroglial dysfunction, or mitochondrial defects are likely to be present. This uncoupling needs further characterization, possibly using hybrid PET/MRI, to establish a mechanistic basis. These observations raise an interesting question of whether focused pharmacological restoration of blood-flow regulation could alleviate symptoms of schizophrenia.

## Data Availability Statement

The individual participant datasets analyzed in this article are not publicly available as we used summary data from published studies. Requests to access the summary data used for the meta-analysis should be directed to LPALANIY@UWO.CA.

## Author Contributions

LP conceived, designed, supervised data collection and statistical analysis, interpreted the results, and drafted the manuscript. NS undertook literature search, collected the data, undertook statistical analysis, and drafted the manuscript. PS supported literature search, cross-checked the clinical data, and drafted the manuscript. UA supported the literature search, oversaw study quality metrics, and drafted the manuscript.

## Conflict of Interest

LP reports personal fees from Otsuka Canada, SPMM Course Limited, UK, Canadian Psychiatric Association; book royalties from Oxford University Press; investigator-initiated educational grants from Janssen Canada, Sunovion and Otsuka Canada outside the submitted work. PS reports personal fees from Otsuka Canada, SPMM Course Limited, UK, outside the submitted work.

The remaining authors declare that the research was conducted in the absence of any commercial or financial relationships that could be construed as a potential conflict of interest.

## References

[1] Beer MD Psychosis: from mental disorder to disease concept. Hist Psychiatry (1995) 6:177–200. 10.1177/0957154X9500602204 11639691

[2] Oliveira ÍAF Guimarães TM Souza RM Dos Santos AC Machado-de-Sousa JP Hallak JEC Brain functional and perfusional alterations in schizophrenia: an arterial spin labeling study. Psychiatry Res Neuroimaging (2018) 272:71–8. 10.1016/j.pscychresns.2017.12.001 29229240

[3] Muoio V Persson PB Sendeski MM The neurovascular unit - concept review. Acta Physiol Oxf Engl (2014) 210:790–8. 10.1111/apha.12250 24629161

[4] Telischak NA Detre JA Zaharchuk G Arterial spin labeling MRI: Clinical applications in the brain. J Magn Reson Imaging (2015) 41:1165–80. 10.1002/jmri.24751 25236477

[5] Miele E Spinelli GP Tomao F Zullo A De Marinis F Pasciuti G Positron Emission Tomography (PET) radiotracers in oncology – utility of 18F-Fluoro-deoxy-glucose (FDG)-PET in the management of patients with non-small-cell lung cancer (NSCLC). J Exp Clin Cancer Res CR (2008) 27:52. 10.1186/1756-9966-27-52 18928537 PMC2579910

[6] Hanson DR Gottesman II Theories of schizophrenia: a genetic-inflammatory-vascular synthesis. BMC Med Genet (2005) 6:7. 10.1186/1471-2350-6-7 15707482 PMC554096

[7] Stanimirovic DB Friedman A Pathophysiology of the neurovascular unit: disease cause or consequence? J Cereb Blood Flow Metab (2012) 32:1207–21. 10.1038/jcbfm.2012.25 PMC3390807 22395208

[8] Watts ME Pocock R Claudianos C Brain Energy and Oxygen Metabolism: Emerging Role in Normal Function and Disease. Front Mol Neurosci (2018) 11:216. 10.3389/fnmol.2018.00216 29988368 PMC6023993

[9] Bachneff SA Regional cerebral blood flow in schizophrenia and the local circuit neurons hypothesis. Schizophr Bull (1996) 22:163–82. 10.1093/schbul/22.1.163 8685659

[10] Liu J Qiu M Constable RT Wexler BE Does baseline cerebral blood flow affect task-related blood oxygenation level dependent response in schizophrenia? Schizophr Res (2012) 140:143–8. 10.1016/j.schres.2012.06.028 PMC3423495 22789669

[11] Walther S Schäppi L Federspiel A Bohlhalter S Wiest R Strik W Resting-State Hyperperfusion of the Supplementary Motor Area in Catatonia. Schizophr Bull (2017) 43:972–81. 10.1093/schbul/sbw140 PMC5581902 27729486

[12] Pinkham AE Liu P Lu H Kriegsman M Simpson C Tamminga C Amygdala Hyperactivity at Rest in Paranoid Individuals With Schizophrenia. Am J Psychiatry (2015) 172:784–92. 10.1176/appi.ajp.2014.14081000 25815418

[13] Ben-Shachar D Bonne O Chisin R Klein E Lester H Aharon-Peretz J Cerebral glucose utilization and platelet mitochondrial complex I activity in schizophrenia: A FDG-PET study. Prog Neuropsychopharmacol Biol Psychiatry (2007) 31:807–13. 10.1016/j.pnpbp.2006.12.025 17329000

[14] Cui LB Chen G Xu ZL Liu L Wang HN Guo L Cerebral blood flow and its connectivity features of auditory verbal hallucinations in schizophrenia: A perfusion study. Psychiatry Res Neuroimaging (2017) 260:53–61. 10.1016/j.pscychresns.2016.12.006 28024236

[15] Horn H Federspiel A Wirth M Müller TJ Wiest R Wang JJ Structural and metabolic changes in language areas linked to formal thought disorder. Br J Psychiatry J Ment Sci (2009) 194:130–8. 10.1192/bjp.bp.107.045633 19182174

[16] Kindler J Jann K Homan P Hauf M Walther S Strik W Static and dynamic characteristics of cerebral blood flow during the resting state in schizophrenia. Schizophr Bull (2015) 41:163–70. 10.1093/schbul/sbt180 PMC4266282 24327756

[17] Kindler J Schultze-Lutter F Hauf M Dierks T Federspiel A Walther S Increased Striatal and Reduced Prefrontal Cerebral Blood Flow in Clinical High Risk for Psychosis. Schizophr Bull (2018) 44:182–92. 10.1093/schbul/sbx070 PMC5768043 28575528

[18] Ota M Ishikawa M Sato N Okazaki M Maikusa N Hori H Pseudo-continuous arterial spin labeling MRI study of schizophrenic patients. Schizophr Res (2014) 154:113–8. 10.1016/j.schres.2014.01.035 24581548

[19] Pinkham A Loughead J Ruparel K Wu WC Overton E Gur R Resting quantitative cerebral blood flow in schizophrenia measured by pulsed arterial spin labeling perfusion MRI. Psychiatry Res (2011) 194:64–72. 10.1016/j.pscychresns.2011.06.013 21831608 PMC3185150

[20] Scheef L Manka C Daamen M Kühn KU Maier W Schild HH Resting-state perfusion in nonmedicated schizophrenic patients: a continuous arterial spin-labeling 3.0-T MR study. Radiology (2010) 256:253–60. 10.1148/radiol.10091224 20505069

[21] Stegmayer K Stettler M Strik W Federspiel A Wiest R Bohlhalter S Resting state perfusion in the language network is linked to formal thought disorder and poor functional outcome in schizophrenia. Acta Psychiatr Scand (2017) 136:506–16. 10.1111/acps.12790 PMC5656821 28865406

[22] Walther S Federspiel A Horn H Razavi N Wiest R Dierks T Resting state cerebral blood flow and objective motor activity reveal basal ganglia dysfunction in schizophrenia. Psychiatry Res (2011) 192:117–24. 10.1016/j.pscychresns.2010.12.002 21511443

[23] Zhu J Zhuo C Qin W Xu Y Xu L Liu X Altered resting-state cerebral blood flow and its connectivity in schizophrenia. J Psychiatr Res (2015) 63:28–35. 10.1016/j.jpsychires.2015.03.002 25812945

[24] Desco M Gispert JD Reig S Sanz J Pascau J Sarramea F Cerebral metabolic patterns in chronic and recent-onset schizophrenia. Psychiatry Res (2003) 122:125–35. 10.1016/S0925-4927(02)00124-5 12714176

[25] Horga G Fernández-Egea E Mané A Font M Schatz KC Falcon C Brain metabolism during hallucination-like auditory stimulation in schizophrenia. PloS One (2014) 9:e84987. 10.1371/journal.pone.0084987 24416328 PMC3885666

[26] Jeong-Hee K Jong-Hoon K YD S YH J SY L HK K Altered interregional correlations between serotonin transporter availability and cerebral glucose metabolism in schizophrenia: A high-resolution PET study using [11C]DASB and [18F]FDG. Schizophr Res (2017) 182:55–65. 10.1016/j.schres.2016.10.020 27760700

[27] Park IH Kim JJ Chun J Jung YC Seok JH Park HJ Medial prefrontal default-mode hypoactivity affecting trait physical anhedonia in schizophrenia. Psychiatry Res (2009) 171:155–65. 10.1016/j.pscychresns.2008.03.010 19217758

[28] Alsop DC Detre JA Golay X Günther M Hendrikse J Hernandez-Garcia L Recommended Implementation of Arterial Spin Labeled Perfusion MRI for Clinical Applications: A consensus of the ISMRM Perfusion Study Group and the European Consortium for ASL in Dementia. Magn Reson Med (2015) 73:102–16. 10.1002/mrm.25197 PMC4190138 24715426

[29] Waxman AD Herholz K Lewis DH Herscovitch P Minoshima S Mountz JM Society of Nuclear Medicine Procedure Guideline for FDG PET Brain Imaging (Version 1.0). Soc Nucl Med (2009) 12.

[30] Radua J Mataix-Cols D Phillips ML El-Hage W Kronhaus DM Cardoner N A new meta-analytic method for neuroimaging studies that combines reported peak coordinates and statistical parametric maps. Eur Psychiatry (2012) 27:605–11. 10.1016/j.eurpsy.2011.04.001 21658917

[31] Sterne JAC Sutton AJ Ioannidis JPA Terrin N Jones DR Lau J Recommendations for examining and interpreting funnel plot asymmetry in meta-analyses of randomised controlled trials. BMJ (2011) 343:d4002. 10.1136/bmj.d4002 21784880

[32] Ioannidis JPA Trikalinos TA An exploratory test for an excess of significant findings. Clin Trials (2007) 4:245–53. 10.1177/1740774507079441 17715249

[33] Albajes-Eizagirre A Solanes A Radua J Meta-analysis of non-statistically significant unreported effects. Stat Methods Med Res (2019) 28:3741–54. 10.1177/0962280218811349 30514161

[34] Radua J Romeo M Mataix-Cols D Fusar-Poli P A general approach for combining voxel-based meta-analyses conducted in different neuroimaging modalities. Curr Med Chem (2013) 20:462–6. 10.2174/0929867311320030017 23157638

[35] Wise T Radua J Nortje G Cleare AJ Young AH Arnone D Voxel-Based Meta-Analytical Evidence of Structural Disconnectivity in Major Depression and Bipolar Disorder. Biol Psychiatry (2016) 79:293–302. 10.1016/j.biopsych.2015.03.004 25891219

[36] Radua J Borgwardt S Crescini A Mataix-Cols D Meyer-Lindenberg A McGuire PK Multimodal meta-analysis of structural and functional brain changes in first episode psychosis and the effects of antipsychotic medication. Neurosci Biobehav Rev (2012) 36:2325–33. 10.1016/j.neubiorev.2012.07.012 22910680

[37] Radua J Grau M van den Heuvel OA Thiebaut de Schotten M Stein DJ Canales-Rodríguez EJ Multimodal voxel-based meta-analysis of white matter abnormalities in obsessive-compulsive disorder. Neuropsychopharmacol. Off. Publ. Am. Coll. Neuropsychopharmacol (2014) 39:1547–57. 10.1038/npp.2014.5 PMC4023155 24407265

[38] Radua J Mataix-Cols D Voxel-wise meta-analysis of grey matter changes in obsessive-compulsive disorder. Br J Psychiatry J Ment Sci (2009) 195:393–402. 10.1192/bjp.bp.108.055046 19880927

[39] Palaniyappan L Liddle PF Does the salience network play a cardinal role in psychosis? An emerging hypothesis of insular dysfunction. J Psychiatry Neurosci (2012) 37:17–27. 10.1503/jpn.100176 21693094 PMC3244495

[40] Menon V Uddin L Saliency, switching, attention and control: a network model of insula function. Brain Struct Funct (2010) 214:655–67. 10.1007/s00429-010-0262-0 PMC2899886 20512370

[41] Li M Li X Das TK Deng W Li Y Zhao L Prognostic Utility of Multivariate Morphometry in Schizophrenia. Front Psychiatry (2019) 10:245. 10.3389/fpsyt.2019.00245 31037060 PMC6476259

[42] Supekar K Cai W Krishnadas R Palaniyappan L Menon V Dysregulated Brain Dynamics in a Triple-Network Saliency Model of Schizophrenia and Its Relation to Psychosis. Biol Psychiatry Immune Mech Psychosis (2019) 85:60–9. 10.1016/j.biopsych.2018.07.020 30177256

[43] Di X Kannurpatti SS Rypma B Biswal BB Calibrating BOLD fMRI Activations with Neurovascular and Anatomical Constraints. Cereb Cortex (2013) 23:255–63. 10.1093/cercor/bhs001 PMC3539449 22345358

[44] Goozée R Handley R Kempton MJ Dazzan P A systematic review and meta-analysis of the effects of antipsychotic medications on regional cerebral blood flow (rCBF) in schizophrenia: association with response to treatment. Neurosci Biobehav Rev (2014) 43:118–36. 10.1016/j.neubiorev.2014.03.014 24690578

[45] Gur RE Turetsky BI Cowell PE Finkelman C Maany V Grossman RI Temporolimbic Volume Reductions in Schizophrenia. Arch Gen Psychiatry (2000) 57:769–75. 10.1001/archpsyc.57.8.769 10920465

[46] Lee SH Niznikiewicz M Asami T Otsuka T Salisbury DF Shenton ME Initial and Progressive Gray Matter Abnormalities in Insular Gyrus and Temporal Pole in First-Episode Schizophrenia Contrasted With First-Episode Affective Psychosis. Schizophr Bull (2016) 42:790–801. 10.1093/schbul/sbv177 26675295 PMC4838098

[47] Crespo-Facorro B Nopoulos PC Chemerinski E Kim JJ Andreasen NC Magnotta V Temporal pole morphology and psychopathology in males with schizophrenia. Psychiatry Res (2004) 132:107–15. 10.1016/j.pscychresns.2004.09.002 15598545

[48] Yi-Chia W Tseng JR Wu C Su FC Weng WC Hsu CC Different FDG-PET metabolic patterns of anti-AMPAR and anti-NMDAR encephalitis: Case report and literature review - Wei - 2020 - Brain and Behavior - Wiley Online Library. Brain Behav (2020) 10:1540. 10.1002/brb3.1540 PMC7066351 31985135

[49] Probasco JC Solnes L Nalluri A Cohen J Jones KM Zan E Decreased occipital lobe metabolism by FDG-PET/CT. Neurol Neuroimmunol Neuroinflamm (2017) 5:413. 10.1212/NXI.0000000000000413 PMC5688263 29159205

[50] Minzenberg MJ Laird AR Thelen S Carter CS Glahn DC Meta-analysis of 41 Functional Neuroimaging Studies of Executive Function in Schizophrenia. Arch Gen Psychiatry (2009) 66:811–22. 10.1001/archgenpsychiatry.2009.91 PMC2888482 19652121

[51] Taylor SF Tandon R Koeppe RA Global cerebral blood flow increase reveals focal hypoperfusion in schizophrenia. Neuropsychopharmacol Off Publ Am Coll Neuropsychopharmacol (1999) 21:368–71. 10.1016/S0893-133X(98)00109-2 10457533

[52] Kuschinsky W Suda S Sokoloff L Local cerebral glucose utilization and blood flow during metabolic acidosis. Am J Physiol Heart Circ Physiol (1981) 241:H772–7. 10.1152/ajpheart.1981.241.5.H772 7304767

[53] Uranova NA Zimina IS Vikhreva OV Krukov NO Rachmanova VI Orlovskaya DD Ultrastructural damage of capillaries in the neocortex in schizophrenia. World J Biol Psychiatry Off J World Fed Soc Biol Psychiatry (2010) 11:567–78. 10.3109/15622970903414188 20109113

[54] Shishido F Uemura K Inugami A Tomura N Higano S Fujita H Cerebral oxygen and glucose metabolism and blood flow in mitochondrial encephalomyopathy: a PET study. Neuroradiology (1996) 38:102–7. 10.1007/BF00604789 8692416

[55] Sano M Ishii K Momose Y Uchigata M Senda M Cerebral metabolism of oxygen and glucose in a patient with MELAS syndrome. Acta Neurol Scand (1995) 92:497–502. 10.1111/j.1600-0404.1995.tb00487.x 8750117

[56] Lourenço CF Ledo A Caetano M Barbosa RM Laranjinha J Age-Dependent Impairment of Neurovascular and Neurometabolic Coupling in the Hippocampus. Front Physiol (2018) 9:913. 10.3389/fphys.2018.00913 30065657 PMC6056650

[57] Das TK Javadzadeh A Dey A Sabesan P Théberge J Radua J Antioxidant defense in schizophrenia and bipolar disorder: A meta-analysis of MRS studies of anterior cingulate glutathione. Prog Neuropsychopharmacol Biol Psychiatry (2019) 91:94–102. 10.1016/j.pnpbp.2018.08.006 30125624

[58] Dempster K Jeon P MacKinley M Williamson P Théberge J Palaniyappan L Early treatment response in first episode psychosis: a 7-T magnetic resonance spectroscopic study of glutathione and glutamate. Mol Psychiatry (2020) 25:1640–50. 10.1038/s41380-020-0704-x PMC7387300 32205866

[59] Kumar J Liddle EB Fernandes CC Palaniyappan L Hall EL Robson SE Glutathione and glutamate in schizophrenia: a 7T MRS study. Mol Psychiatry (2020) 25:873–82. 10.1038/s41380-018-0104-7 PMC7156342 29934548

[60] Albajes-Eizagirre A Radua J What do results from coordinate-based meta-analyses tell us? Neuroimage (2018) 176:550–3. 10.1016/j.neuroimage.2018.04.065 29729389

