# Supplementary material for: Neurovascular Uncoupling in Schizophrenia: A Bimodal Meta-Analysis of Brain Perfusion and Glucose Metabolism
Source: Front Psychiatry. 2020 Aug 5;11:754. doi: 10.3389/fpsyt.2020.00754 (PMC7427579; doi:10.3389/fpsyt.2020.00754)
Supplement: Supplementary file 1 [file DataSheet_1.docx]

**Neurovascular uncoupling in schizophrenia: A bimodal meta-analysis of brain perfusion and glucose metabolism**

**SUPPLEMENTARY MATERIAL**

**Table S1.** rCBF or rCMRglu abnormalities in schizophrenia: meta-regression analyses

|  | MNI  Coordinates | SDM  z-value ^(a)^ | *P* Value ^(b)^ | No. of  voxels ^(c)^ | Breakdown  (No. of voxels) ^(c)^ |
| --- | --- | --- | --- | --- | --- |
|  |  |  |  |  |  |
| *EFFECTS OF ILLNESS DURATION* |  |  |  |  |  |
| *Decreased rCBF or rCMRglu in patients with longer illness duration*  *(high < low duration)* |  |  |  |  |  |
| Left inferior frontal gyrus (triangular) | -52,42,-2 | -2.118 | 0.00001 | 125 | Left BA45  Left BA46  Left BA47 |
| *EFFECTS OF ANTIPSYCHOTIC MEDICATION* |  |  |  |  |  |
| *Increased rCBF or rCMRglu in patients receiving higher doses of antipsychotics*  *(high > low doses)* |  |  |  |  |  |
| Right middle occipital gyrus | 36, -92, 6 | 2.638 | <0.0001 | 47 | Right BA18 |
| Left striatum | -22, 0, 2 | 1.912 | <0.0001 | 586 | Left BA48 |
| *EFFECTS OF NEGATIVE SYMPTOM SEVERITY* |  |  |  |  |  |
| *Decreased rCBF or rCMRglu in patients with higher PANSS negative scores*  *(high < low severity)* |  |  |  |  |  |
| Right middle occipital gyrus | 38, -92, 2 | -3.839 | <0.0001 | 88 | Right BA18 |
| Left superior occipital gyrus | -20, -90, 28 | -3.307 | <0.0001 | 86 | Left BA18 |

(a) Voxel probability threshold: p = 0.0005 (difference in slope)

(b) Peak height threshold: z = 1

(c) Cluster extent threshold: 10 voxels. Regions with less than 10 voxels are not reported in the cluster breakdown.

**Table S2.** Combined rCBF and rCMRglu abnormalities in schizophrenia: multimodal analyses

|  | MNI  Coordinates | *P* Value ^(b)^ | No. of  voxels ^(c)^ | Breakdown  (No. of voxels) ^(c)^ |
| --- | --- | --- | --- | --- |
|  |  |  |  |  |
|  |  |  |  |  |
| *Increased rCBF with reduced rCMRglu*  *Reduced rCBF with increased rCMRglu*  *Increased rCBF with Increased rCMRglu*  Left striatum  Right striatum  Right inferior temporal gyrus  Left temporal pole | none  none  -28,-2,-12  26,-4,-6  44,4,-40  -  38,-2,-44 | <0.001  <0.001  <0.001  <0.001 | 1146  1022  535  490 | Left BA34  Left BA48  Right BA34  Right BA48  Right BA20  Right BA36  Right BA20  Right BA36 |
| *Reduced rCBF with reduced rCMRglu* |  |  |  |  |
| Right median cingulate | 6,22,34 | <0.001 | 2115 | Bilateral BA32  Bilateral BA24  Bilateral BA8  Left BA9 |
| Left inferior frontal (triangular) | -44,22,0 | <0.001 | 606 | Left BA47  Left BA45  LeftBA48  Left BA38 |
|  |  |  |  |  |

(a) Voxel probability threshold: p = 0.0025 (tolerable pre-conjunction threshold p=0.05; error in p estimate assumed with desired threshold p<0.001)

(b) Peak height threshold: p=0.00025

(c) Cluster extent threshold: 10 voxels. Regions with less than 10 voxels are not reported in the cluster breakdown.

**Figure S3.** Correlation between patient antipsychotic dosage and negative symptom severity across studies included in the main analysis

**Figure S4.** Funnel plots for (a) Right putamen, and (b) Right median cingulate gyrus

**
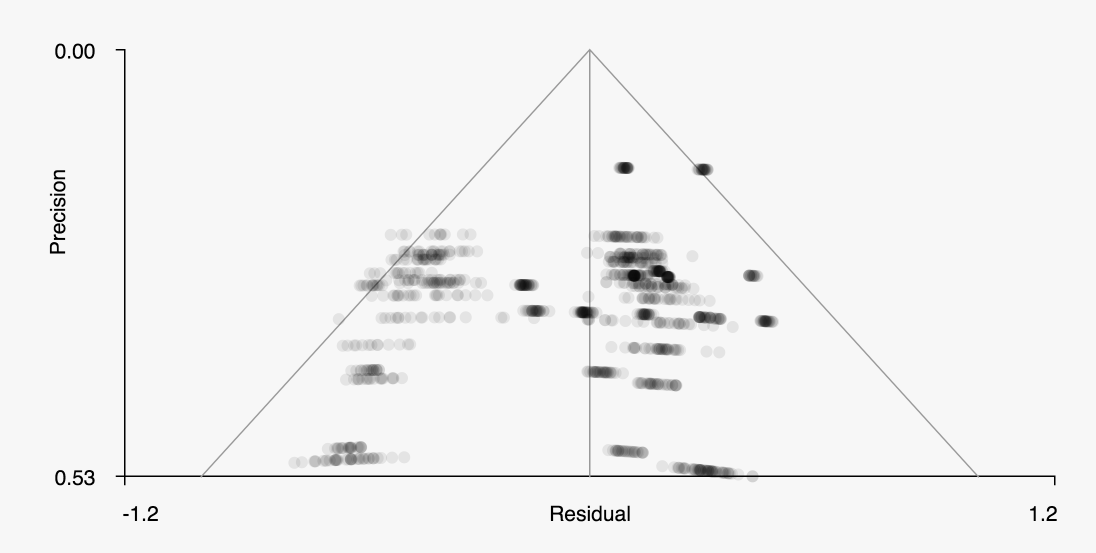
**

**(a**) Right Putamen (Bias test: z=-0.67, p=0.502; Excess significance test: p=0.86; I^2^ statistic: I^2^=3.47)

**
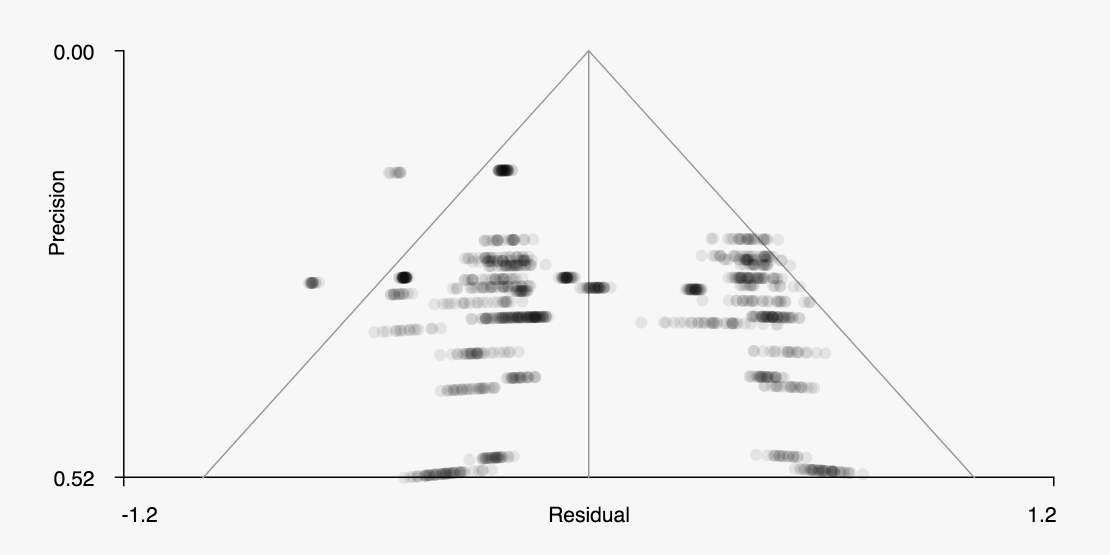
**

**(b)** Right Median Cingulate Gyrus (Bias test: z=0.62, p=0.538; Excess significance test: p=0.49; I^2^ statistic: I^2^=30.1)

| **ASL studies** | **Reference Test** | **MRI Scanner** | **Field Strength** | **Label Type** | **Label Duration/TI1 (ms)** | **PLD/TI (ms)** | **Readout** | **Total Scan Time or # Control and Label pairs** | **T1 Blood (ms)** | **Label Efficiency** | **Motion Correction** | **CBF Quantification** | **Spatial Normalization Template** | **Intensity Scaling** | **Exclusion of non-GM Voxels** | **Accounted for Multiple Comparison Problem** |
| --- | --- | --- | --- | --- | --- | --- | --- | --- | --- | --- | --- | --- | --- | --- | --- | --- |
| **Cui_2017** | DSM-IV, PANSS, Prodromal Questionnaire confirm absence of psychotic syndrome in HC | Siemens Trio | 3.0T | PASL-PICORE |  | 1800 |  | 4 min 20 s |  |  | Yes | Yes | PET Template | NO | No | AlphaSim (p<0.001, cluster extent >13 for cluster P<0.05) |
| **Horn_2009** | DSM-IV based on Clinical interview, PANSS, Scale for the Assessment of Thought, Language and Communication | Siemens Vision | 1.5 T | PASL-QUIPPS II | 700 | 1400 |  | NS | 1200 | 0.9 | Yes | Yes |  | No | No | Cluster-size thresholding in BrainVoyager QX 1.7.6 |
| **Kindler 2015** | Clinical Intreview and ICD-10, psychiatry history, PANSS, PsyRats, AHRS | Siemens Trio | 3.0T | pCASL | 1600 | 1250 |  | 5 mins 44 s |  |  | Yes | Yes |  | No | No | familywise Error (FWE) correction |
| **Kindler 2018** | ICD-10, PANSS, PsyRats | Siemens Trio | 3.0T | pCASL | 1600 | 1250 |  | NS |  |  | Yes | Yes | SPM MNI | Global Proportional | Yes | familywise Error (FWE) correction (small volume) |
| **Liu2012** | DSM-IV | Siemens | 3.0T | PASL-QUIPSS II | 1400 | 1700 |  | NS |  |  | NS | Yes | MNI | No | No | Partial correction Cluster-size thresholding |
| **Oliveria_2018** | DSM-V, PANSS | Philips Achieva | 3.0T | pCASL | 1620 | 1525 | 2D EPI | 6 min 48s | 1650 | 0.85 | Yes | Yes | MNI | Global GM mean | PVE-Asllani | FDR Correction |
| **Ota_2014** | DSM-IV,PANSS | Philips | 3.0T | pCASL | 1650 | 1520 | 2D EPI | 4 min 24 s | 1664 | 0.85 | NS | Yes | MNI T1 | No | ANCOVA | No |
| **Pinkham 2015** | DSM-IV with Structured Clinical Interview, PANSS | Philips Achieva | 3.0T | pCASL | 1650 | 1525 |  | 40 | 1279 | 0.86 | Yes | Yes | MNI | Global GM mean | No | AlphaSim p<0.05 |
| **Pinkham 2011** | Diagnostic Interview for Genetic studies, Best Estimate Final Diagnoses, SANS, SPAS | Siemens Trio | 3.0T | PASL-QUIPPS II | 700 | 1900 | 2D EPI | 40 | 1600 | 0.97 | Yes | Yes | MNI | No |  | AlphaSim p<0.05 |
| **Scheef 2010** | DSM-IV, PANSS | Philips Achieva | 3.0T | CASL | 2200 | 800 | 2D EPI | 40 | 1490 | 0.68 | Yes | Yes | PET Template | Global scaling | No | FDR p<0.05 |
| **Stegmayer 2017** | mini international neuropyschiatric interview, clinical interviews, PANSS | Siemens Trio | 3.0T | pCASL | 1600 | 1500 |  | 55 | 1650 | 0.95 | Yes | Yes | MNI T1 | Global GM mean ACOVA |  | TFCE (FWE) p<0.05 |
| **Walther 2011** | DSM-IV, PANSS | Siemens Trio | 3.0T | PASL-QUIPPS II | 700 | 1000 |  | 5 mins 9s | 1490 | 0.95 | Yes (3D PACE) | Yes | NS | No | No | FDR q<0.01 |
| **Walther 2017** | DSM-V, CASH, PANSS | Siemens Trio | 3.0T | pCASL | NS | NS |  | 55 |  |  | Ns | Yes | Ns | No | No | NO |
| **Zhu 2015** | DSM-IV, PANSS | GE Discovery MR750 | 3.0T | pCASL | NS | 2025 | 3D FSE | 4min 44s |  |  | Ns | Yes | PET Template | Global mean | No | FWE p<0.05 |

**Table S5.** ASL acquisition parameter data

| **PET studies** | **Reference Test** | **PET Scanner** | **Dose (Mbq)** | **Uptake Period (min)** | **Scan Duration (min)** | **Fasting (minimum 4 hours)** | **Uptake Environment** | **Recon Method** | **Motion Compensation** | **PET Correction Mentioned** | | | | | **Intensity Scaling** | **Spatial Normalization Template** | **Exclusion of non-GM Voxels** | **Accounted for Multiple Comparison Problem** |
| --- | --- | --- | --- | --- | --- | --- | --- | --- | --- | --- | --- | --- | --- | --- | --- | --- | --- | --- |
|  |  |  |  |  |  |  |  |  |  | **Attenuation** | **Scatter** | **Dead** | **Random** | **Decay** |  |  |  |  |
| **Ben_Shachar 2006** | DSM-IV, PANSS, Clinical Global Impression scale | Standalone PET; PET Positron Corp HZL/R | 370 | 30 | 15 |  | Yes | Filtered back projection |  | Yes | Yes | Yes | Yes |  | Global | SPM MNI | Yes | Unclear |
| **Desco 2003** | DSM-IV, SANS, SPAS SCID confirmed interviews | Standalone PET; Positron Posicam EZL | 370 | 20 |  | 6 | Yes; eyes open ears unplugged |  |  |  |  |  |  |  | Global ANCOVA | Talairach | Yes | Peak-height corrected p<0.05, extent-corrected p<0.05, uncorrected p<0.001 |
| **Horga 2014** | DSM-IV Structred Clinical Interview, PANSS and CASH | Standalone PET; Advance Nxi, GE Healthcare | 4.7/Kg | 35 | 20 | 4 | dark room | Iterative | Head Immbolized | Yes |  |  |  |  | Global Proportional | SPM MNI |  | P<0.05 FWE or P<0.05 FDR |
| **Kim 2017** | DSM-IV Structured Clinical Interview, PANSS | Standalone PET;Siemens HRRT | 172-255 | 40 | 30 |  | eyes closed; dark room | Iterative |  | Yes |  |  |  | Yes | Cerebellum | SPM MNI |  | P<0.05 FWE |
| **Park 2009** | DSM-IV Structred Clinical Interview and PANSS | Standalne PET; GE Advance | 185 | 40 | 15 |  | Yes (eyes closed) | Filtered back projection |  | Yes |  |  |  |  | Global Proportional | PET Template |  | peak height threshold uncorrected p<0.0005 extent threshold corrected p<0.05 |

**Table S6.** PET acquisition parameter data
